# Supplementary material for: Functional Cyperus esculentus L. Cookies Enriched with the Probiotic Strain Lacticaseibacillus rhamnosus SL42
Source: Foods. 2024 Aug 15;13(16):2541. doi: 10.3390/foods13162541 (PMC11353252; doi:10.3390/foods13162541)
Supplement: Supplementary file 1 [file foods-13-02541-s001.zip › foods-3141480-supplementary.pdf]

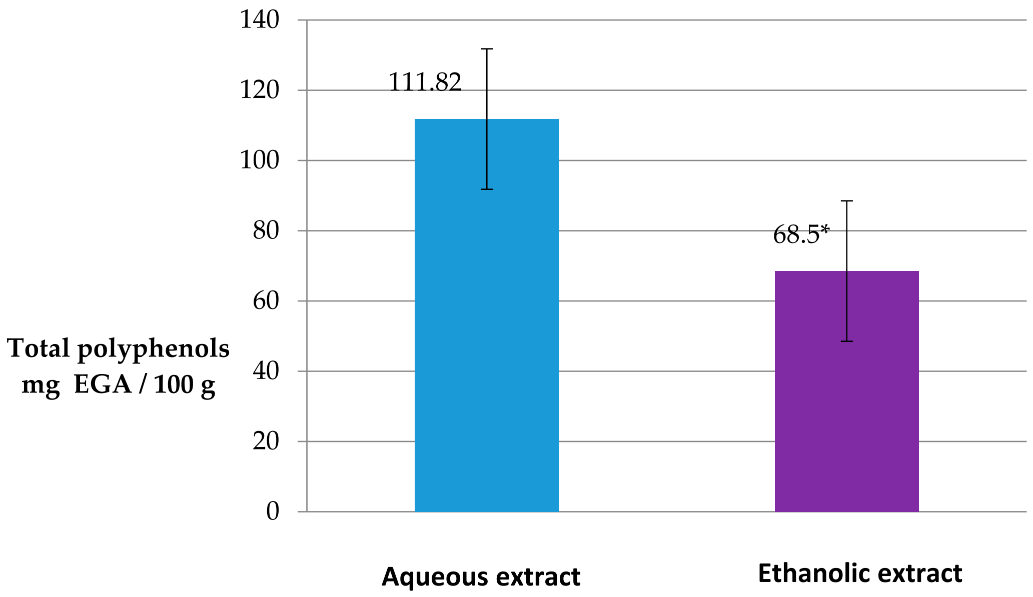

Figure S1. Total polyphenol contents in aqueous and ethanolic extracts of tiger nut. *(P<0.05).

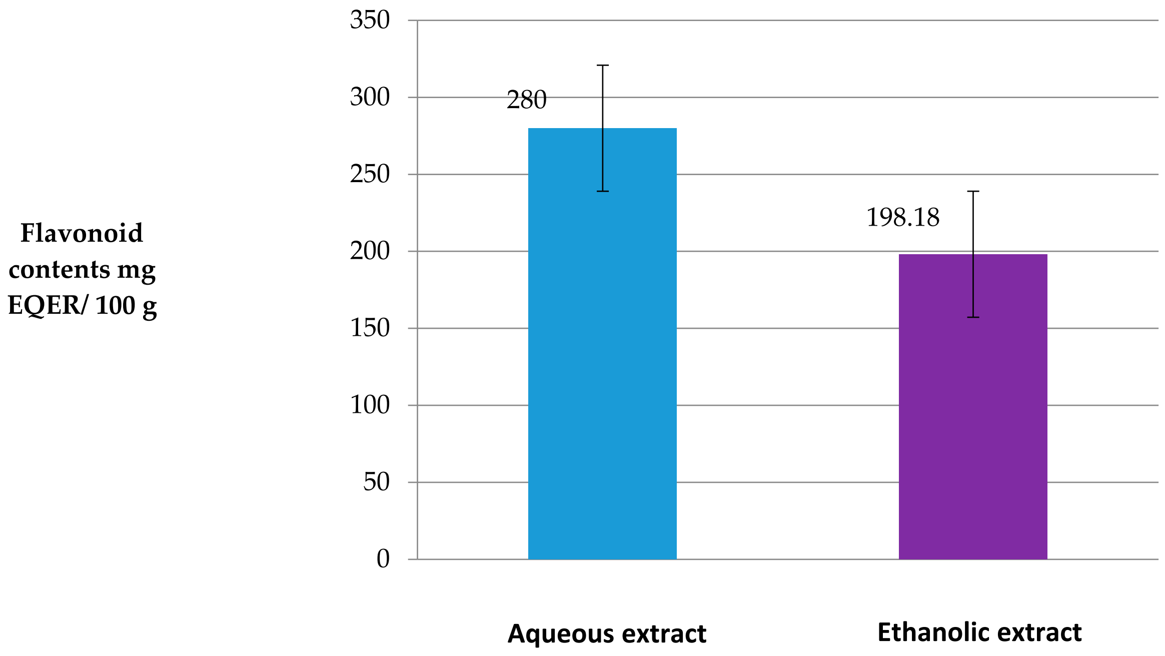

Figure S2. Flavonoid contents in aqueous and ethanolic extracts of tiger nut.

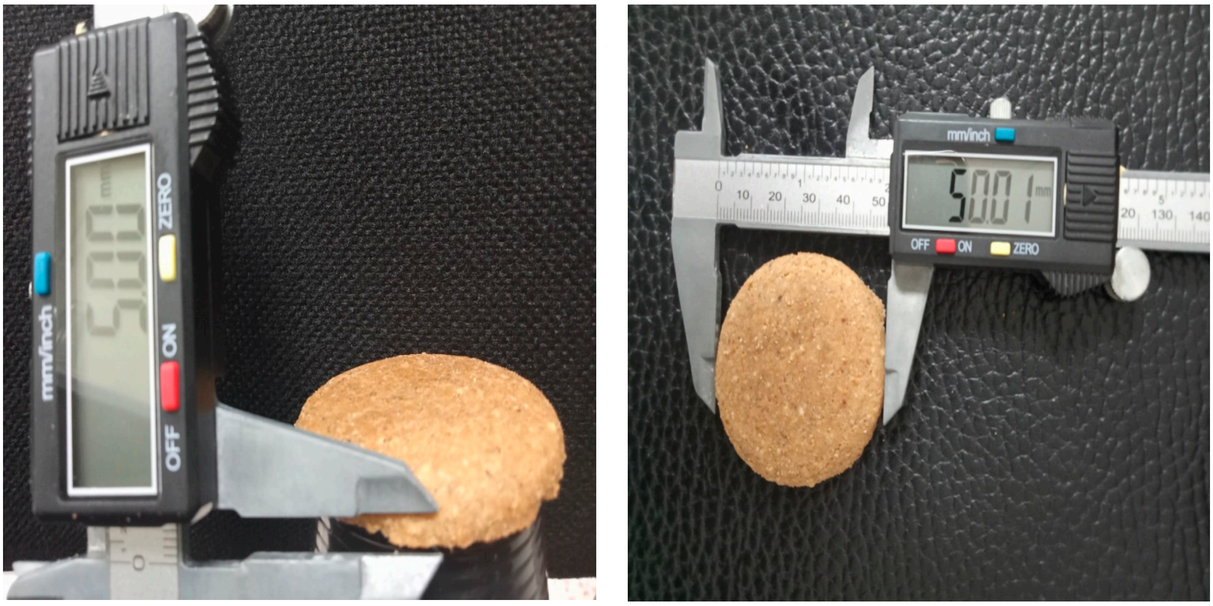

Figure S3. Diameter, thickness of the baked cookies**.**

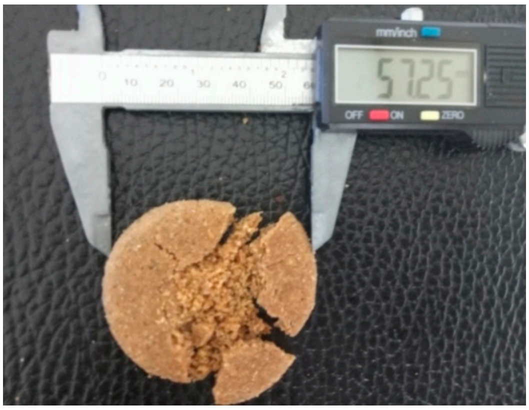

Control tiger nut cookie (CC)

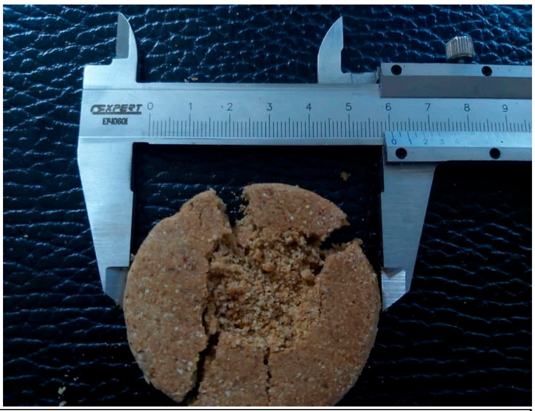

Tiger nut cookie with honey-syrup (CH)

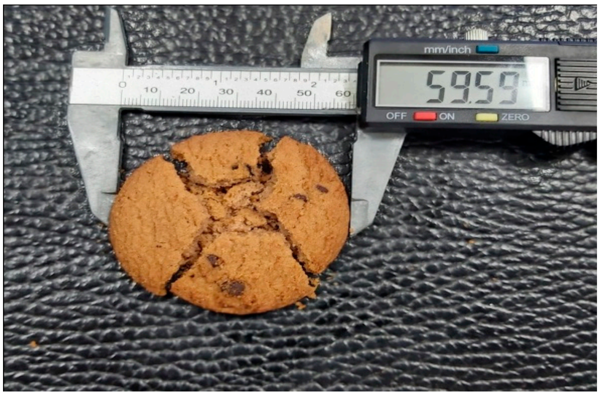

Commercial cookie

Figure S4. Assessment of the cookie's vulnerability to break (mm).

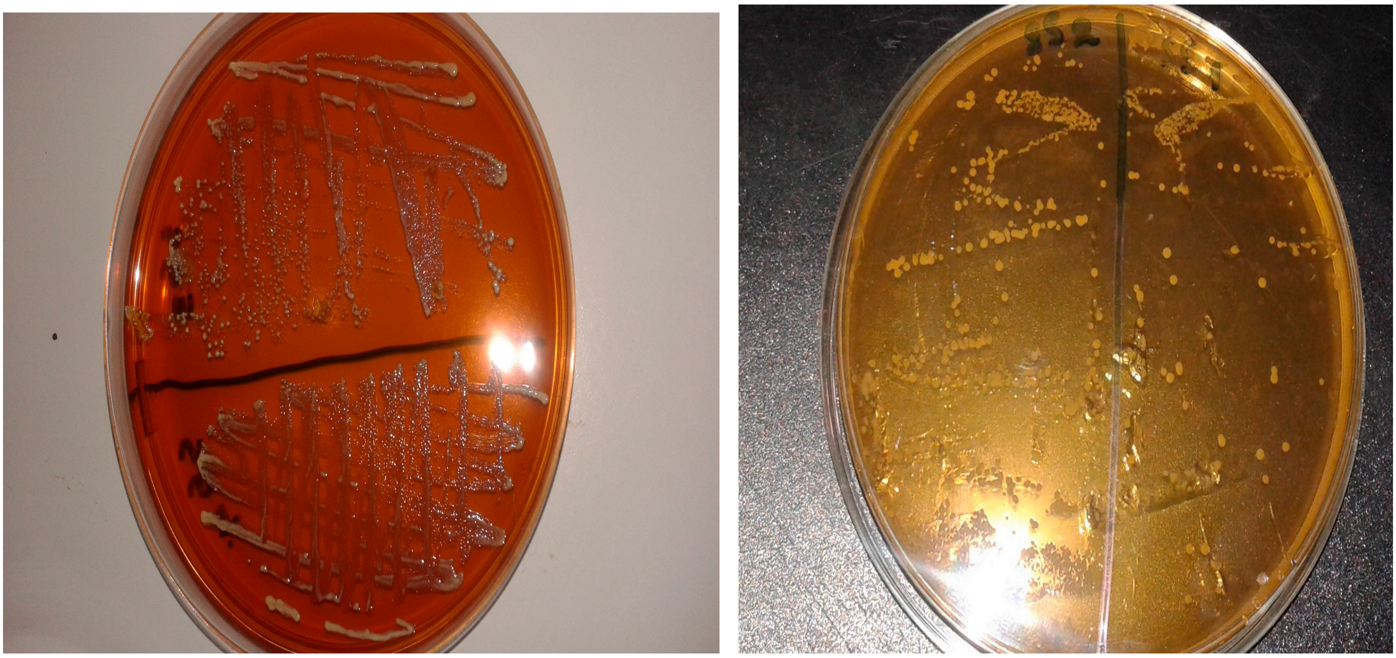

SL42 in the presence of 2% tiger nut flour

SL42 in the presence of 5% honey-bee

Figure S5. SL42's growth tests in the presence of tiger nut flour (2%, w/v) or honey-bee (5%, w/v).

Table S1. Antimicrobial activity[1] of tiger nuts.

Pathogens	Inhibition zone (mm)	
	Aqueous extract	*Hydro-ethanolic extract (30v/70v)*
Candida albicans ATCC 10231	10 ± 0.18b	25 ± 0.36a
Escherichia coli ATCC 25922	11 ± 0.02b	23 ± 0.10a
Bacillus cereus ATCC 10876	12 ± 0.11a	16 ± 0.10a
Staphylococcus aureus ATCC 33862	17 ± 0.30b	24 ± 0.20a
Pseudomonas aeruginosa ATCC 27853	08 ± 0.22b	22 ± 0.20a
Bacillus subtilis ATCC 6051	11 ± 0.08b	19 ± 0.04a

[1] The results are means of three independent replicates (n = 3) ± SD.

[a-b] : Significant differences in the same row.

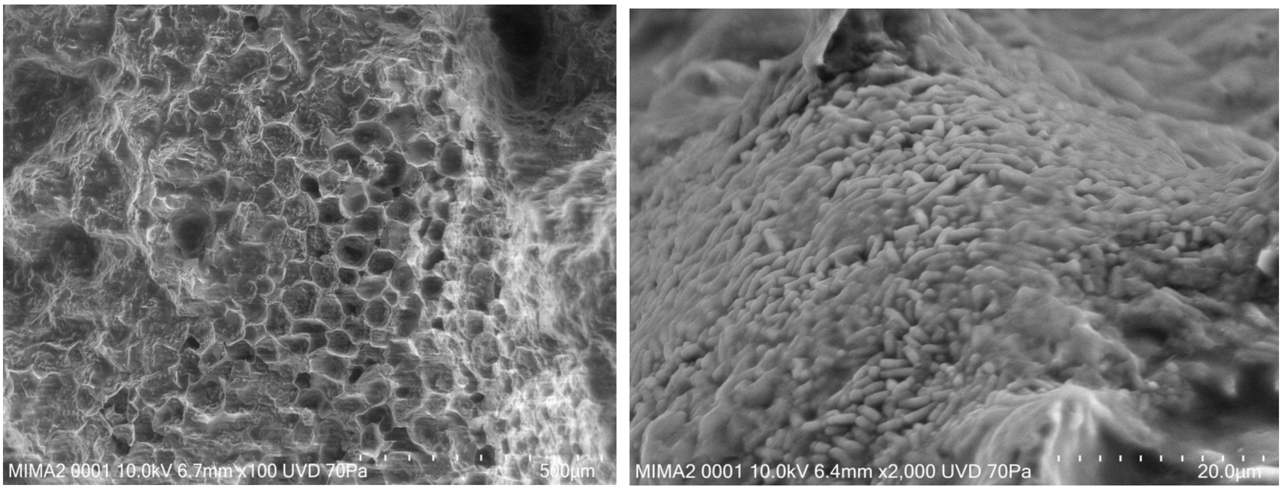

Figure S6. SEM environmental mode analysis of the unfixed cookies (CH) enriched with honey-bee syrup containing *L. rhamnosus* SL42 at the 7th day storage.
